# The impact of use of an intraoperative margin assessment device on re-excision rates

**DOI:** 10.1186/s40064-015-0801-5

**Published:** 2015-04-28

**Authors:** Molly Sebastian, Stephanie Akbari, Beth Anglin, Erin H Lin, Alice M Police

**Affiliations:** Virginia Hospital Center, Reinsch Pierce Family Center for Breast Health, 1625 N George Mason Dr, Arlington, VA 22205 USA; Medical Center of Plano, Complete Breast Care, 3801 W. 15th Street Building A, Suite 210, Plano, TX 75075 USA; Department of Surgery, University of California Irvine Medical Center, 333 City Blvd. West, Suite 1600, Orange, CA 92868 USA

## Abstract

Historically there has been a high rate of surgical interventions to obtain clear margins for breast cancer patients undergoing breast conserving local therapy. An intraoperative margin assessment tool (MarginProbe) has been approved for use in the US since 2013. This study is the first compilation of data from routine use of the device, to assess the impact of device utilization on re-excision rates. We present a retrospective, observational, review from groups of consecutive patients, before and after the implementation of intraoperative use of the device during lumpectomy procedures. Lesions were localized by standard methods. The intraoperative margin assessment device was used on all circumferential margins of the main specimen, but not on any additional shavings. A positive reading by the device led to an additional shaving of the corresponding cavity location. Specimens were also, when feasible, imaged intra-operatively by X-ray, and additional shavings were taken if needed based on clinical assessment. For each surgeon, historical re-excision rates were established based on a consecutive set of patients from a time period proximal to initiation of use of the device. From March 2013 to April 2014 the device was routinely used by 4 surgeons in 3 centers. In total, 165 cases lumpectomy cases were performed. Positive margins resulted in additional re-excision procedures in 9.7% (16/165) of the cases. The corresponding historical set from 2012 and 2013 consisted of 186 Lumpectomy cases, in which additional re-excision procedures were performed in 25.8% (48/186) of the cases. The reduction in the rate of re-excision procedures was significant 62% (P < 0.0001). Use of an intraoperative margin assessment device contributes to achieving clear margins and reducing re-excision procedures. As in some cases positive margins were found on shavings, future studies of interest may include an analysis of the effect of using the device on the shavings intra-operatively.

## Background

Breast conservation surgery followed by radiation is the recommended treatment (National Institute of Health 2014) for most early stage (I & II) breast cancer. From recent reports, in the US a breast conserving approach amounts to 60-75% of the procedures for treatment of early stage breast cancer (Katipamula et al. 2009; Cancer Trends Progress Report – 2011). This treatment approach has been shown to be equivalent to treatment by mastectomy (Veronesi et al. 2002; Fisher et al. 2002). With the implementation of screening programs and the advancement of diagnostic techniques, these constitute the majority of diagnosed breast cancers.

Obtaining clear margins following a lumpectomy surgery for breast cancer is an important factor in the treatment of breast cancer while conserving the breast (BCS). This is not a trivial task, and in many cases the margins are not clear following the initial lumpectomy procedure, necessitating a re-excision in order to achieve clear margins. From population based studies the rates of re-excision procedures in the US are reported to be approximately 25% (McCahill et al. 2012; Morrow et al. 2009). There have been numerous reports and discussions of the topic on clear margins, including meta analyses (Houssami et al. 2010; Wang et al. 2012; Blair et al. 2009; Dunne et al. 2009). Recently, this topic has been the subject of an updated SSO/ASTRO guideline (Moran et al. 2014).

MarginProbe (MP, by Dune Medical Devices, Paoli, PA, USA) is a new device developed to identify cancerous tissue at the margins of excised lumpectomy specimens. The device is used intraoperatively, in real-time, enabling immediate margin resection. The device measures the local electrical properties of breast tissue, and provides a positive/negative reading for each measurement (7 mm diameter). The surgeon interrogates the surface of the lumpectomy specimen. The device has been evaluated in several multicenter trials, see review in (Thill et al. 2013), where patients were randomized to standard of care techniques for margin evaluation versus standard of care with additional (adjunctive) use of MP. These studies have shown that adjunctive use of MP leads to a > 50% reduction in the rate of re-excision procedures. These studies also showed that the device integrates well with the OR work flow, and that cosmetic outcomes were not affected. MP performance is the same for the different cancer histology subgroups, including DCIS. MP has been approved for use in the US since the beginning of 2013.

Here we provide the first report on routine use of MP in 3 centers in the US. The effect on the rate of re-excision procedures was based on comparison to historical records.

## Methods

Retrospective review observational (routine-data-based) study at 3 centers, 4 surgeons, all using their routine lumpectomy methods, both before, and after initiation of use of MP. No standardization between surgeons was required. Criteria for re-excision were per surgeon/institution. MP was used on a routine basis, without any patient selection criteria. All patients were recently diagnosed with breast cancer.

Mammographic/Ultrasound findings were typically histologically confirmed via biopsy. Lesions were, in general, wire localized. Specimens were oriented and margin borders were delineated. In accordance with general practice, standard Intra-operative assessment included palpation, x-ray, ultra-sound and gross pathology, whichever was appropriate for a given patient. MarginProbe was used as an adjunctive tool for intra-operative margin assessment on the main lumpectomy specimen, following its excision. All 6 faces/aspects were interrogated (excluding skin and fascia/muscle). Duration of use is 3 to 5 minutes. Additional shavings were taken based on device readings. All specimens were processed by routine permanent pathology.

### Data collection

Data was collected for the period from when each center/surgeon started using the device up until April 2014. Data was collected from operative notes, pathology reports & hospital records. Data on device readings was obtained from operative notes. Margins status was also recorded for the main lumpectomy specimen. For each center, historical data were obtained for time periods of duration similar to that in which MP was used. Historical data were obtained from the years 2012 and 2013, time periods proximal (shortly before) to the initiation of use of the device. As this was a retrospective review observational study, there was no need for consent.

### Statistical analysis

Numerical variables were tabulated using mean, standard deviation, and ranges. Categorical variables were tabulated using number of observations and percent. All statistics were performed at α = 0.05 two-sided significance level. Rates between arms were compared using Fisher’s exact test. No missing data was imputed.

## Results

Up until April 2014 the device was used on a total 165 patients. Table 1 shows the break-down of patients per surgeon, and the time at which each center started using the device. The earliest being March 2013.

**Table 1:** **Device utilization**

**Surgeon**	**Started using MP**	**Procedures performed**	**Procedures per month**
1	May-13	39	4.3
2	May-13	42	3.8
3	Nov-13	39	7.9
4	Mar-13	45	3.5
Total		165	

Table 2 describes patient baseline characteristics. The distributions of age, tumor type histology, and receptor status data are consistent with those reported in the 2011 SEER database (Howlader et al. 2014). Patients diagnosed with DCIS amounted to 20%, very similar to the 2011 SEER database value of 20.4%.

**Table 2:** **Patient demographics**

Age			
Mean (STD)		60.5 (12.4)	
<40		5	3%
40 to 50		30	18%
50 to 60		46	28%
60 to 70		44	27%
>70		39	24%
Tumor type			
Invasive Ductal		119	72%
Invasive Lobular		12	7%
DCIS		33	20%
Other (Sarcoma met)		1	1%
Receptor status	Pos	Neg	% Pos
ER	99	12	89%
PR	84	27	76%
HER2	15	89	14%

As seen in Table 3, most all patients were diagnosed by core biopsy. Most lesions were localized pre-surgery, consistent with them being early stage cancer patients. Only 21% the patients had palpable tumors.

**Table 3:** **Pre-operative**

Pre-operative diagnosis by:		
core biopsy	163	98.8%
open biopsy	1	0.6%
no biopsy	1	0.6%
Lesion localization:		
Needle Localization (FNL)	127	77%
Ultrasound clip	4	2%
None (palpable)	34	21%

Tumor characteristics based on final pathology are presented in Table 4. Average tumor size was 1.5 (+/- 1.1) cm, with the majority of the lesions (76%) less than 2 cm in size. In the majority of the invasive tumors, 59% (77/131) the tumor had a DCIS component. Overall, there was DCIS present in 67% of tumors.

**Table 4:** **Tumor characteristics (based on final pathology)**

Tumor composition		
IDC	37	22.4%
DCIS	32	19.4%
IDC + DCIS	75	45.5%
ILC	11	6.7%
ILC + DCIS	1	0.6%
IPC	2	1.2%
ITC	4	2.4%
ITC + DCIS	1	0.6%
PCIS	1	0.6%
Metastatic sarcoma	1	0.6%
Tumor grade:		
Invasive grade		
1	41	35%
2	56	47%
3	21	18%
In-Situ grade		
1	20	20%
2	52	51%
3	29	29%
Tumor/lesion size (cm)		
Mean (STD)	1.5 (1.1)	
<1	51	35%
1 to 2	58	40%
>2	35	24%

IDC: Invasive Ductal Carcinoma; ILC: Invasive Lobular Carcinoma; DCIS: Ductal Carcinoma In-situ; IPC: Invasive Papillary Carcinoma; ITC: Invasive Tubular Carcinoma; PCIS: Papillary Carcinoma In-situ.

For 127 of the cases pre-operative tumor type composition was available (in the operative notes and/or pathology reports). These data are presented in Table 5. Pre-operatively, the tumor composition was invasive, with no DCIS component, in 50% (64/125) of the cases. For these, when comparing to final pathology, in 56% (36/64) of the cases a DCIS component was identified on final pathology. Out of the 34 cases pre-diagnosed as DCIS, 15% (5/34) were upstaged to Invasive cancer on final pathology.

**Table 5:** **Comparison to pre-operative tumor type composition diagnosis**

IDC	64	50%
IDC + DCIS	15	12%
DCIS	34	27%
ILC	4	3%
other	10	8%
Total	127	
Based on final path		
DCIS added	36	56%
Upstage to Invasive	5	15%

To evaluate the contribution of MP in reducing the rate of re-excision procedures, the re-excision rates with use of MP were compared to those from a corresponding historical set. For each surgeon, the historical set consisted of a consecutive series of cases from a time period shortly before she started using MP. The set was from a duration of time similar to that for which MP was in use, ensuring that the fraction of cases (for each surgeon) in the historical set will be distributed between surgeons in the same manner as the cases in which MP was used. As seen in Table 6, the total number of cases in the historical set and the number of cases per surgeon are similar to those where MP was used.

**Table 6:** **Historical control/set comparison of re-excision procedures**

		**Total**	**Per Surgeon**			
			1	2	3	4
**MarginProbe Procedures**	Lumpectomy procedures	165	39	42	39	45
	Re-excision procedures	16	3	7	4	2
	Re-excision rate	9.7%	8%	17%	10%	4%
**Historical**	Lumpectomy procedures	186	51	48	46	41
	Re-excision procedures	48	8	21	14	5
	Re-excision rate	25.8%	16%	44%	30%	12%
**Reduction**	Absolute Reduction (% points)	16.1%	8.0%	23.8%	20.2%	7.8%
	Relative reduction	62%	51%	62%	66%	64%
	P-value	<0.0001				

The numbers and rates of re-excision procedures are also presented in Table 6, both for the MP cases and the historical set. With use of MP, in 9.7% (16/165) of the cases there was a re-excision procedure performed. In the historical set, in 25.8% (48/186) of the cases there was a re-excision procedure performed. The relative reduction in the rate of re-excision procedures was 61%, with the corresponding absolute reduction being 16.1%. The reduction was highly statistically significant, P < 0.0001.

As is also seen from Table 6, the reduction was similar across surgeons. The re-excision rates for the historical set varied by more than a factor of 3, spanning from 12% to 44%. The per surgeon relative reduction in the re-excision rates was between 51% and 66%. The re-excision rates with use of MP were between 4% and 17%. Note that criteria for performing a re-excision were not standardized between surgeons.

Re-excision procedures are performed due to insufficient margin clearance based on final pathology. As the device was used only on the main specimen, it could only contribute to the identification of margins on the initial lumpectomy specimen. As a result, in some cases, even though there were shavings taken, the margins of the shavings themselves remained persistently involved, leading to a re-excision procedure. From Table 7, out of the total re-excision procedure rate of 9.7%, 6.1% (10/165) of the re-excision procedures were due to failed detection of margins on main specimen. The remaining 3.6% (6/165) were due to involved margins on shavings.

**Table 7:** **Break-down of additional surgical procedures**

Re-Excision Lumpectomy procedures	9.7% (16/165)
Due to failed detection of margins on main specimen	6.1% (10/165)
Due to margins on shavings	3.6% (6/165)
Mastectomy as a second procedure (due to extensive disease)	1.8% (3/165)

An additional situation may occur when there was an underestimation of the extent of the disease pre-surgery. As presented in Table 7, there were 1.8% (3/165) cases in which mastectomy was performed as a second procedure; this being due to the identification on final pathology of extensive disease with insufficient margin clearance. Cancer was also present within the shavings taken.

## Discussion

Surgical treatment of breast cancer while conserving the breast is a recommended, and well accepted approach for the treatment of early stage (stage I & II) breast cancer. Associated with this approach is the need to perform re-excision procedures in order to achieve negative margins. A significant fraction of the women treated by breast conserving surgery will undergo additional surgeries to achieve clear margins. Intraoperative assessment of the lumpectomy specimen is routinely performed, but standard techniques such as palpation and specimen imaging (by radiography and/or ultrasound), are not able to discern microscopically residual disease at the margins. This limitation is more pronounced in the case of non-palpable lesions and DCIS (Balch et al. 2005; Jeevan et al. 2012). Intraoperative margin assessment with specimen imaging and immediate pathologic analysis (Cabioglu et al. 2005) and immediate excision of any margins thought to be close or positive can reduce the need for a return to the operating room. This approach requires close coordination between surgery, pathology, and diagnostic radiology and may not be possible to implement in all practice settings.

The MP device provides real-time intraoperative assessment of microscopic lumpectomy margins. Published reports on use of the device have so far been in the settings of clinical studies. In this retrospective case review, we provided the first report on routine use of the device in the USA. The device integrated very well with the workflow in the operating room, taking no more than 5 minutes to use. There were no adverse events associated with device use. Adjunctive use of the device significantly decreases, when compared to corresponding historical series, by 62% the rate of patients who required re-excision procedures. The margin thresholds for returning a patient to the operating room were not standardized between surgeons. Still, this relative reduction was similar irrespective of the nominal rates per surgeon. With use of the device, the rate of re-excision procedures was 9.7%.

Most all of the patients in this contemporary consecutive series were early stage breast cancer patients, with 79% of the malignant lesions localized prior to surgery. The majority of the tumors (67%) included an intra-ductal (DCIS) component. Additionally, there was a significant number of cases where DCIS was only identified post-surgery. This contributes to the challenge the surgeon faces with regards to margins.

Identifying the involved margin and the extent of the malignant lesion(s) intra-operatively may also assist in identifying extensive disease that was not picked up during the cancer diagnosis. There were 3 patients in which extensive disease (within the shavings as well) was identified, who proceeded directly to mastectomies.

There is no complete correlation between positive margins status and the presence of cancer during re-excision procedures (Skripenova & Layfield 2010; Scopa et al. 2006). With use of the device there were 28 cases in which the margin on the main specimen was clear, but the corresponding shaving taken contained cancer. These correspond to disease that would not have been identified during final pathology, had not the shavings been taken. This may reflect the presence of discontinues disease, or may be due to the sampling limitation inherent to the histopathology processing methods.

The topic of what constitute a clear margin has long been under debate. Lately, there have been renewed discussions related to this topic (Houssami et al. 2014; Jagsi et al. 2014; Hunt & Sahin 2014; Buchholz et al. 2014; Hunt et al. 2014). Specifically, the new SSO guidelines (Moran et al. 2014) suggest using tumor on ink (tumor cells at the cut surface) as the definition for positive margin. In view of this, we also looked at the contribution of device in identification of positive margins on ink. In 18% (30/165) of the cases the primary (main) lumpectomy specimen, prior to intra-operative assessment, had tumor on ink. In 73% (22/30) of these cases with use of device the positive margins were identified. In 3.6% (6/165) of the cases re-excision procedures were performed due to margins on ink. Of these, only 2.4% (4/165) were due to failed detection of tumor cells at the margins.

Re-excision procedures also represent a significant burden to the medical system. According to the National Cancer Institute, in 2008, 60% of women age 20 and older diagnosed with early-stage breast cancer underwent breast-conserving surgery (Cancer Trends Progress Report – 2011). According to American Cancer Society, 296980 cases of female breast cancer, both invasive and DCIS, were diagnosed in 2013 (American Cancer Society 2013). If 60% of these patients underwent breast conserving procedures, there would be 178188 lumpectomy procedures performed. A re-excision rate of 25% would translate to 44547 re-excision procedures. Utilizing the MarginProbe could decrease the re-excision rate by 62%, resulting in 27612 fewer of these surgeries. There would be significant savings to the health care system resulting from this decrease in the re-excision rate, and the burden to the patient would be reduced.

The inability to achieve acceptable lumpectomy margins with one operative procedure, has multiple negative effects. It increases the burden to the patient, who is subject to the inconvenience, discomfort and heightened anxiety of the unexpected second procedure and the loss of productive time from work and family. Additional surgeries carry an added risk of complication and negatively impact cosmetic outcomes (Heil et al. 2012). The necessity for additional surgery may also lead to a delay in the initiation of other required therapies.

The fear and uncertainty generated by the potential need for a re-excision procedure may be related to the recent increase (King et al. 2011) in the proportion of patients with early stage breast cancer opting for mastectomy. Additionally, in some cases, patients “refuse” the recommendation for a re-excision procedure, putting them in a higher risk for in breast reoccurrence.

The device was used on the lumpectomy specimen, but not on additional shavings taken. About a third (6/16) of the re-excision procedures were due to the shavings being positive. It would be interesting to investigate how to incorporate device use on the shavings as well.

Utilization of techniques for partial breast irradiation and intraoperative breast irradiation (Vaidya et al. 2010) are also negatively impacted by re-excision procedures. The importance of obtaining clear margins is elevated when performing Onco-plasitc procedures during the initial lumpectomy surgery, as it may not be as clear where additional tissue should be removed. And the additional surgery could negatively impact the cosmetic result.

## Conclusions

This report on routine use of MarginProbe provides additional support for use of the device. Use of the device was associated with a significant decrease in the rate of re-excision procedures. This provides the surgeons with added confidence when performing lumpectomy surgery, reduces the burden for the health care system, and increases the assurance of patients in this surgical treatment approach for breast cancer.
